# A Case of Two Diametrically Opposed Gastric Xanthomas in the Pyloric Antrum: An Unusual and Benign Endoscopic Finding

**DOI:** 10.7759/cureus.65296

**Published:** 2024-07-24

**Authors:** Christos Sotiropoulos, Georgios J Theocharis, Konstantinos C Thomopoulos

**Affiliations:** 1 Gastroenterology, University General Hospital of Patras, Patras, GRC

**Keywords:** gastric xanthelasmas, foamy histiocytes, yellowish mucosal plaques, gastrointestinal endoscopy, gastric xanthomas

## Abstract

Gastrointestinal tract xanthomas are rarely encountered findings in upper gastrointestinal endoscopy. They are non-tumor, well-demarcated, yellowish mucosal plaques most commonly diagnosed in the stomach, especially in the antrum or the pyloric region. Histologically, a gastric xanthoma consists of foamy histiocytes in the lamina propria. Although it is a benign lesion, it can be associated with various precancerous conditions and its appearance can resemble malignancy.

We report the case of a 74-year-old female patient who presented to the outpatient clinic with a six-month history of intermittent pain in the epigastrium. The patient’s physical examination as well as the hematological and biochemical investigations were normal. The upper gastrointestinal endoscopy revealed yellowish plaques in the pyloric antrum of the stomach, which were diagnosed as gastric xanthomas by histological examination.

The significance of gastric xanthomas in relation to gastric disease still remains to be determined. Given the frequent association of gastric xanthomas and known premalignant lesions and occasionally malignant changes of the gastric mucosa, we need to pay close attention to endoscopic diagnosis and histochemical and immunohistochemical evaluation.

## Introduction

Gastrointestinal xanthomas or xanthelasmas are uncommon benign lesions that can be found along the gastrointestinal tract, more often in the esophagus, stomach, and duodenum, while small and large intestinal xanthomas are rarely encountered [1-3]. The reported incidence of gastric xanthomas is 0.018-7%, with a higher incidence in women [1,3]. The stomach, antrum, and pyloric regions are the most commonly affected areas [1].

Gastric xanthomas, also known as lipid islands, are benign, well-demarcated, yellowish-white mucosal plaques or nodules characterized by an accumulation of lipids in histiocytic foam cells [2,3]. They can be suspected by their characteristic gross appearance and confirmed by histological examination [1]. Their appearance can resemble gastric malignancies such as signet ring cell-type adenocarcinomas or clear cell-type carcinoid tumors; therefore, histological evaluation of biopsied specimens is recommended to differentiate these conditions [1,2].

Histologically, gastric xanthelasmas are characterized by varying amounts of foamy histiocytes, inflammatory cells, fibrous reactions, and sometimes multinucleated giant cells [4]. The typical histologic appearance of xanthomas consists of clusters of foamy histiocytes containing lipids [2].

The clinical significance and pathogenesis of gastrointestinal xanthomas is not clear [1]. Several studies have suggested that they may result from an underlying inflammatory response to mucosal damage and aging [3]. However, other developmental mechanisms are yet to be elucidated [3]. In some cases, they can be associated with dyslipidemias and diabetes mellitus, while often they can coexist with various precancerous conditions, such as gastrointestinal anastomosis, atrophic gastritis, and *Helicobacter pylori*-associated gastritis, chronic gastritis, and gastric dysplasia [1,4].

Due to their benign nature, gastric xanthomas have received little attention and have been considered of little clinical significance, so far. It remains unclear whether gastric xanthomas would be useful markers for the presence of early gastric cancer and additional investigation is needed for this.

## Case presentation

A 74-year-old Caucasian, non-diabetic, hypertensive female patient presented to outpatient clinics with a six-month history of intermittent vague epigastric pain with dyspepsia, without any other accompanying symptoms. She denied any hyperlipidemia history, alcohol consumption, or non-steroidal anti-inflammatory drug use. Her physical examination was unremarkable, and hematological and biochemical lab values were normal. Her lipid profile was normal (total cholesterol: 177 mg/dL, triglycerides: 65 mg/dL, high-density lipoprotein: 42 mg/dL, and low-density lipoprotein: 79 mg/dL).

**Figure 1:**
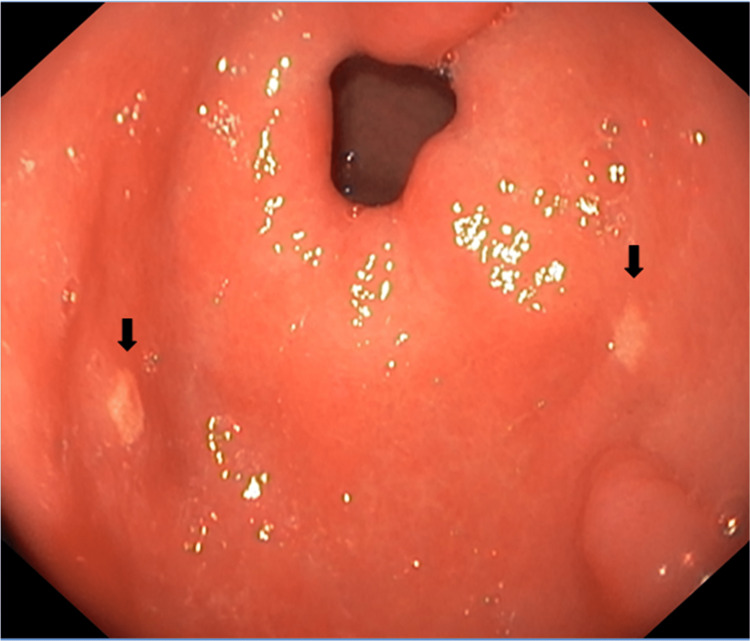
Upper gastrointestinal endoscopy: erythema and edema of the mucosa with two diametrically opposed, slightly elevated, yellowish-white plaque lesions (2-3 mm) in the pyloric antrum (black arrows).

The upper gastrointestinal endoscopy revealed erythema and edema of the mucosa of the stomach, with two diametrically opposed, slightly elevated, yellowish-white plaque lesions (2-3 mm) in the pyloric antrum (Figure 1). The esophagus and duodenum were normal.

**Figure 2:**
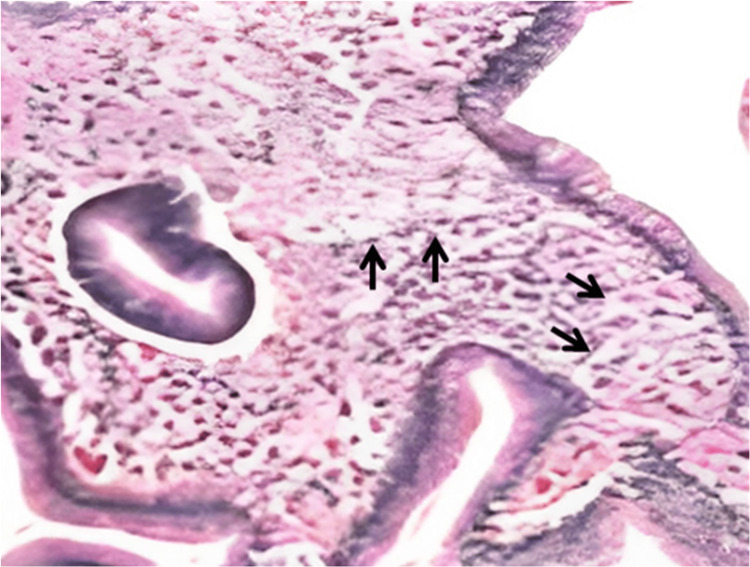
Biopsy specimens: multiple histiocytes with foamy cytoplasms (hematoxylin and eosin staining) (black arrows).

Biopsies of the lesions revealed multiple histiocytes with foamy cytoplasms in the lamina propria (Figure 2), and biopsies from the surrounding area showed chronic inflammatory changes suggestive of chronic gastritis.

Staining with periodic acid-Schiff and chromogranin A was negative, while *Helicobacter pylori* and intestinal metaplasia were not detected. The histochemical and immunohistochemical investigations ruled out the presence of carcinoma cells and confirmed the diagnosis of gastric xanthomas.

The patient was prescribed omeprazole 20 mg/day and was followed up after eight weeks presenting with clinical remission of the pain. A review endoscopy showed persistence of the antral xanthomas but with endoscopic improvement of gastritis.

## Discussion

Gastric xanthomas or lipid islands, localized non-tumor accumulations of foamy histiocytes in the lamina propria of the gastric mucosa, are occasionally observed in upper gastrointestinal endoscopy and are more prevalent with increasing age [1,5]. They are mostly found in the antrum and pylorus and can be single or multiple [1,2].

Data on the frequency of gastric xanthomas, their clinical significance and etiology, and their link with other non-malignant and malignant lesions are significantly different in the published literature [1,2]. It is believed that the mucosa affected by certain pathological processes is more susceptible to their occurrence, and it has been proposed that the transformation of macrophages into foamy cells can be the result of phagocytosis of *Helicobacter pylori*, which penetrate into the lamina propria [2].

Although gastric xanthomas are benign lesions, they may have a deceiving appearance, so histological investigation is considered mandatory [1]. Occasionally, their gross appearance can resemble gastric malignancies, but histology and immunohistochemistry can differentiate these conditions from xanthomas [1].

In our case, xanthomas were diagnosed in an elderly female patient in the absence of associated etiologic factors during the investigation of epigastric pain. Our patient had two diametrically opposed xanthomas in the pyloric antrum, which were observed during an esophagogastroduodenoscopy, and further histological evaluation confirmed the endoscopic finding by the presence of a classical cluster of foamy macrophages in the subepithelial region. Biopsies taken from the surrounding area showed the presence of chronic gastritis. This association has been reported in several case reports and case studies. Other premalignant conditions associated with xanthomas, such as atrophic gastritis, gastrointestinal anastomosis, and gastric dysplasia, were absent in our case. Diabetes mellitus and hyperlipidemia may be coexisting conditions but were not present in our patient. Finally, *Helicobacter pylori* infection was not detected in our patient.

Atrophic gastritis and intestinal metaplasia, basically induced by *Helicobacter pylori*, are well-established endoscopic risk indicators of gastric cancer [6]. The association of gastric xanthomas with *Helicobacter pylori* infection and atrophic gastritis suggests the hypothesis that the existence of these benign lesions might correlate with future gastric cancer development [6]. Therefore, the endoscopic finding of this uncommon and harmless lesion is important. Present data on gastric cancer investigation suggest that the presence of gastric xanthomas in patients with chronic and atrophic gastritis may serve as an effective warning sign alerting physicians to the possible development of early gastric cancer and the need for surveillance [3].

The natural course of gastrointestinal xanthomas is not yet clearly determined and they can disappear without any intervention [1]. In some cases, endoscopic mucosal resection has been successfully performed, mostly in the presence of associated polyps, while endoscopic argon plasma coagulation treatment and heat probe therapy have also demonstrated good results [1]. As xanthelasmas are usually incidental findings and benign conditions, regular follow-up is advised only if alarming symptoms develop or there is a high index of suspicion of malignancy [1]. In our patient, we decided to follow up on the case.

## Conclusions

Despite gastric xanthomas being benign lesions, they can be deceiving, making histopathological confirmation mandatory. Because xanthomas are frequently associated with known premalignant lesions or occasionally coexist with cancerous changes, it is advisable to perform histochemical and immunohistochemical investigations to confirm the diagnosis and eliminate the presence of gastric cancer. Once xanthelasma is detected, it may serve as a useful predictive endoscopic marker for monitoring gastric cancer. However, further investigation is needed to confirm the utility of these factors.

## References

[1] Dhakal M, Dhakal OP, Bhandari D, Gupta A (2013). Gastric xanthelasma: an unusual endoscopic finding. BMJ Case Rep.

[2] Andrejić BM, Božanić SV, Solajić NS, Djolai MA, Levakov AM (2012). Xanthomas of the stomach: a report of two cases. Bosn J Basic Med Sci.

[3] Sekikawa A, Fukui H, Maruo T, Tsumura T, Kanesaka T, Okabe Y, Osaki Y (2014). Gastric xanthelasma may be a warning sign for the presence of early gastric cancer. J Gastroenterol Hepatol.

[4] Houissa F, Bouslama K, Debbeche R, Mouelhi L, Bouzaidi S, Salem M, Najjar T (2013). Gastric xanthelasma: an uncommon lesion. Tunis Med.

[5] Shibukawa N, Ouchi S, Wakamatsu S, Wakahara Y, Kaneko A (2021). Gastric xanthoma is correlated with early gastric cancer of previously Helicobacter pylori-infected gastric mucosa. JGH Open.

[6] Yamashita K, Suzuki R, Kubo T (2019). Gastric xanthomas and fundic gland polyps as endoscopic risk indicators of gastric cancer. Gut Liver.

